# Amyloid Fragmentation and Disaggregation in Yeast and Animals

**DOI:** 10.3390/biom11121884

**Published:** 2021-12-15

**Authors:** Vitaly V. Kushnirov, Alexander A. Dergalev, Alexander I. Alexandrov

**Affiliations:** Bach Institute of Biochemistry, Federal Research Center “Fundamentals of Biotechnology” of the Russian Academy of Sciences, 119071 Moscow, Russia; alexanderdergalioff@gmail.com

**Keywords:** amyloid, prion, chaperone, amyloid fragmentation, Sup35, α-synuclein, Hsp40, Hsp70, Hsp104, HSP110

## Abstract

Amyloids are filamentous protein aggregates that are associated with a number of incurable diseases, termed amyloidoses. Amyloids can also manifest as infectious or heritable particles, known as prions. While just one prion is known in humans and animals, more than ten prion amyloids have been discovered in fungi. The propagation of fungal prion amyloids requires the chaperone Hsp104, though in excess it can eliminate some prions. Even though Hsp104 acts to disassemble prion fibrils, at normal levels it fragments them into multiple smaller pieces, which ensures prion propagation and accelerates prion conversion. Animals lack Hsp104, but disaggregation is performed by the same complement of chaperones that assist Hsp104 in yeast—Hsp40, Hsp70, and Hsp110. Exogenous Hsp104 can efficiently cooperate with these chaperones in animals and promotes disaggregation, especially of large amyloid aggregates, which indicates its potential as a treatment for amyloid diseases. However, despite the significant effects, Hsp104 and its potentiated variants may be insufficient to fully dissolve amyloid. In this review, we consider chaperone mechanisms acting to disassemble heritable protein aggregates in yeast and animals, and their potential use in the therapy of human amyloid diseases.

## 1. Introduction

Amyloids are filamentous protein aggregates with regular cross-beta type structure. They are generated from the soluble form of a single protein through self-catalytic structural conversion. Amyloid formation is regarded to be the cause of more than forty incurable age-associated diseases, including Alzheimer’s disease and Parkinson’s disease (PD) [1]. Due to their self-catalytic nature, some amyloids can be infectious, and, in this case, they are called prions, i.e., proteinaceous infectious agents. Only one protein in humans and animals, PrP, is known to form a bona fide prion that is able to infect other organisms naturally [2]. However, many amyloids are able to spread within an organism, or in certain tissues, thus showing prion-like properties [3,4], and there is emerging evidence that some can also be infectious via direct injection [5]. Not all amyloids discovered in animals are associated with pathologies. Some of them serve useful functions, such as amyloids of the proteins CPEB of *Aplysia* or Orb2 of *Drosophila*, which are involved in the formation of long-term memory [6,7].

Several amyloids with prion properties were found in yeast [8]. The first yeast prion, [*PSI*^+^], was discovered by Brian Cox in 1965 as an unusual nonsense-suppressor phenotype with non-Mendelian genetic properties [9]. This phenotype segregated 4:0 in crosses of [*PSI*^+^] with non-prion [*psi*^−^] cells; it could be spontaneously lost or cured with certain chemicals, as well as appearing de novo [10]. Another genetic element with similar behavior, [*URE3*], which allows the utilization of poor nitrogen sources in the presence of rich ones, was discovered by Francois Lacroute in 1971 [11]. However, their prion nature was explained only in 1994 by Reed Wickner, who related them to the prion-like conversion of the Sup35 and Ure2 proteins [12]. Further studies revealed several other yeast proteins that formed amyloid prions: Rnq1, Swi1, Cyc8, Mod5, Mot3, and Nup100 [13,14,15,16,17,18]. Amyloidogenic domains of more than ten other proteins form prions when fused to the Sup35 functional domain, though the propagation in a prion mode was not shown for these proteins on their own [17]. Transient prion-like amyloids can be induced by heat stress [19,20], and non-heritable functional amyloids called mnemons have been shown to mediate yeast cellular memory [21]. 

Of note, there are many prions that are apparently not based on the amyloid structure, like the [*GAR*+] prion, which allows overriding glucose-associated repression of alternative carbon source utilization [22]. A recent groundbreaking systematic search revealed about 46 novel yeast prions, most of which relied on some type of protein aggregation of a poorly characterized, seemingly non-amyloid, type [23]. 

Yeast prions are usually not highly detrimental to cells and can even increase the ability of a yeast population to adapt to changing environmental conditions [23,24], though some prion-associated phenotypes are not necessarily beneficial [25]. Yeast amyloid prions proved to be a very efficient model for understanding the basic principles of the formation and propagation of amyloids and prions. Though many specific manifestations of mammalian amyloids and prions cannot be reproduced in yeast, this organism is very convenient for the study of their basic properties due to the ease of all kinds of biological manipulations. Moreover, yeast is used as a model to study the aggregation and toxicity of many human and animal amyloidogenic proteins [26,27,28]. 

Yeast studies were central in the formulation of the concept of amyloid fragmentation, the process by which amyloid fibrils are split into smaller pieces through interactions with chaperones. Fragmentation is strictly required for amyloid propagation as a prion [29]. Fragmentation also strongly affects intraorganismal propagation and properties of non-prion amyloids, since smaller amyloids are more mobile and may be more toxic. Here, we review existing data on how chaperones work to disassemble amyloids in yeast and animals, resulting in either amyloid dissolution or prion-like propagation, and the potential of chaperone-based mechanisms in therapies for human amyloid diseases and PD in particular. 

## 2. The Sup35 Protein and Its Prion Structures

### 2.1. Sup35 Protein Function and Architecture

The majority of yeast studies described below involve [*PSI*^+^], the most studied yeast prion, which relates to the prion state of the Sup35 protein. Sup35, also known as translation termination factor eRF3, terminates protein synthesis in cooperation with the Sup45 protein (eRF1) [30,31]. The reduction of Sup35 function due to mutations or prion formation results in an increased readthrough of nonsense codons. This may be conveniently detected as a phenotypic reversion of nonsense mutations. Either one of the two nonsense mutations in the adenine synthesis pathway, *ade1-14* or *ade2-1*, are usually used for the observation of nonsense codon readthrough. Such mutants are unable to grow in the absence of adenine, but they re-acquire this ability when Sup35 converts to its prion state and only a small fraction of it remains soluble and functional. Moreover, *ade* nonsense mutants produce red colonies due to the accumulation of a red intermediate of adenine biosynthesis, while wild-type colonies are white. Depending on the levels of Sup35 that remain soluble/functional, the restoration of adenine biosynthesis can be nearly complete or partial, which results in white or pink colonies. 

Sup35 is composed of three distinct domains. The C-terminal domain (C, residues 254–685) is homologous to the translation elongation factor eEF-1A and is essential for translation termination and cell viability. The N-terminal (N, 1–123) and middle (M, 124–253) domains are non-essential, non-conservative in sequence, and are natively unfolded [32]. The N domain is required for prion properties and forms prion structures. It is rich in glutamine, asparagine, and tyrosine, as is typical of many yeast prion domains. The M domain has no clear function, though it was reported to interact with Hsp104 [33] and to modulate the formation of Sup35 liquid droplets under stress conditions [34]. Lack of the M domain strongly reduces Sup35 levels ([17] and our unpublished observations) via an unknown mechanism. The M domain is rich in the charged residues of lysine and glutamic acid, which is atypical of yeast prion domains.

### 2.2. [PSI^+^] Prion Variants

Similar to many other amyloids and prions [35,36], Sup35 can form many different amyloid structures (variants), which manifest as different phenotypes in vivo [37,38]. [*PSI*^+^] variants are usually distinguished by the level of the nonsense codon readthrough and the stability of inheritance. These phenotypes usually correlate with each other, where higher readthrough corresponds to higher stability. However, such phenotypes do not allow one to reliably distinguish between more than two or three [*PSI*^+^] variants, since these phenotypes can vary slightly depending on the cellular background and so cannot be defined with sufficient precision. In particular, two other prions, [*SWI*^+^] and [*PIN*^+^], can significantly affect the nonsense readthrough by decreasing levels of the eRF1 (Sup45) translation termination factor and can even produce a detectable nonsense-suppressor phenotype in the absence of [*PSI*^+^] [39]. To overcome this restriction, King et al. proposed to distinguish [*PSI*^+^] variants based on their interaction with a panel of Sup35 N domain mutants and GFP fusions [40]. This allowed the authors to confidently distinguish 23 [*PSI*^+^] variants [41]. Three more variants were able to propagate in the presence of the weakened T160M mutant of the Hsp104 chaperone, but not wild-type Hsp104 [42]. In addition, some Sup35 amyloid structures can be obtained in vitro, but cannot stably propagate in vivo [41,43]. All this gives us an idea of how many different folds a single amyloidogenic protein can acquire. 

Notably, only five of the mentioned 23 [*PSI*^+^] variants can be obtained by the standard procedure used in the majority of publications, i.e., Sup35 overproduction in the wild-type background. The additional [*PSI*^+^] variants were obtained by passing these “basic” [*PSI*^+^] variants to various Sup35 mutants and back [41]. The basic [*PSI*^+^] variants were usually roughly divided into two categories. “Strong” [*PSI*^+^] variants showed a high level of nonsense codon readthrough due to very low levels of soluble non-prion Sup35. “Weak” variants exhibited moderate readthrough related to observable levels of soluble Sup35. According to Huang and King, only one of the basic [*PSI*^+^] variants is strong, while the four others are weak [41]. Compared to weak prions, strong ones have smaller fibrils due to better fragmentation [44]. 

Interestingly, some tests reveal a sharp (rather than gradual) difference between the strong and weak [*PSI*^+^] variants. The strong variant is not cured by the multicopy *HSP104* gene and is lethal in the presence of multicopy *SUP35*, while weak isolates are readily curable and non-toxic under these conditions [45].

### 2.3. Sup35 Prion Structures

**Figure 1:**
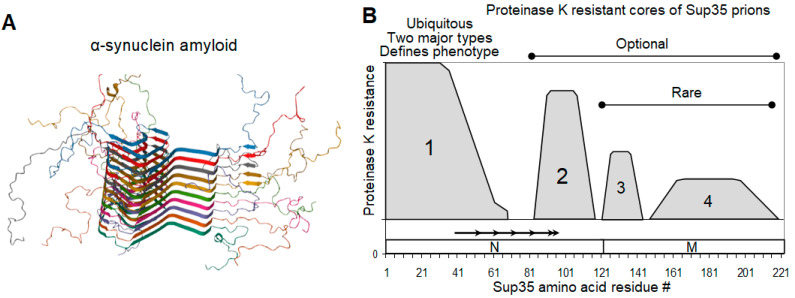
α-synuclein amyloid and Sup35 prion structure. (**A**) A typical parallel in-register amyloid structure exemplified by that of α-synuclein (PDB 2N0A) [48]. (**B**). Map of proteinase K-resistant cores of Sup35 prions. The picture summarizes data for 26 [*PSI*^+^] isolates differing in their origin and phenotype [45]. The location of Sup35 N and M domains, as well as of the imperfect oligopeptide repeats, is shown.

Most [*PSI*^+^] phenotypic variants are likely to have a structural basis in the manner in which monomers are folded in the amyloid fibril. While a full spatial structure of the Sup35 prion has not yet been obtained, it is known that it is a parallel in-register cross-beta structure based on intermolecular beta sheets [46,47]. In this structure, each Sup35 molecule (protomer) occupies a layer of 0.48 nm along the fibril axis, which corresponds to the standard interstrand distance in a beta sheet. Such layers have a uniform thickness, though they are not necessarily perfectly flat, and they may be regarded as a one strand-thick two-dimensional structure. A good example of such a structure is α-synuclein (α-syn) amyloid [48] (Figure 1A). The strain variations in such structures may be due to two partly dependent parameters: (a) the difference in the exact regions involved in the beta structure and (b) the different folding within a layer. The former parameter can be defined by several different methods, including fluorescent labels [49], hydrogen/deuterium exchange (HD) [50], and the resistance to proteinase K (PK) [45,50]. These data largely agree with each other, though with some reservations. Fluorescent labeling with pyrene maleimide (297 Da) and acrylodan (225 Da) indicated that region 1–20 is loosely structured and does not form intermolecular contacts [49]. This contradicts other works and is likely to be an artifact, since [*PSI*^+^] is highly sensitive to single amino acid changes in this region [40,51], while the addition of fluorescent labels with considerable masses may cause an even greater structural perturbation. Therefore, we will describe the Sup35NM prion structure based on our PK mapping data, since they (1) were obtained with ex vivo Sup35 prions, rather than in vitro-generated Sup35 amyloids used in other works, and (2) were made for 26 [*PSI*^+^] isolates differing in their phenotypes and origins [45]. 

The Sup35NM region can contain up to four separate protease-resistant structures, called Cores 1 to 4, located strictly within four regions of residues 2–72, 73–124, 125–153, and 154–221 (Regions 1 to 4, Figure 1B). As the only exception, the structure spanning residues 90 to 143 was observed infrequently. However, only the N-terminal of these structures (Core 1) was present in all [*PSI*^+^] isolates. The majority of weak [*PSI*^+^] variants have a Core 2 structure at residues 91-121, while a few strong [*PSI*^+^] variants have a Core 2 at residues 82–100. Structures in the regions 125–153 and 154–221 belonging to the M domain were present in a small proportion of [*PSI*^+^] isolates. All four structures together were observed only once in the W8 [*PSI*^+^] variant [52]. Of note, some [*PSI*^+^] variants were likely heterogenous for Cores 2 to 4. This implies the simultaneous presence of Core 2 at locations 82–100 and 91–119, and/or, presumably, the presence of any one of the Cores 2 to 4 in only a part of the prion population. This agrees with electron microscopic observations of the structural heterogeneity of Sup35 fibrils seeded in vitro by [*PSI*^+^] lysates [53], and can complicate future detailed structural studies.

To our knowledge, Sup35 is the only amyloid for which a structure composed of more than one part was reported. However, a multipart structure could be present in Rnq1 (our unpublished data) and other yeast prions with large glutamine- and asparagine-rich prion domains.

Surprisingly, the [*PSI*^+^] nonsense-suppressor phenotype seems to be defined solely by apparently small differences in Core 1 structure, rather than by the presence or absence of the other Cores. Core 1 can be further divided into two parts, of which part A (2–32) is fully resistant to PK, while part B (33–72) is only partially resistant and shows lower resistance in weak [*PSI*^+^] [45]. 

The Sup35 N domain is known to contain five imperfect oligopeptide repeats with the sequence PQGGYQ(Q)QYN. Despite being rich in glutamine, tyrosine, and glycine residues typical of yeast prions, the repeats tend to be unstructured or acquire partial PK resistance as a lateral part of Core 1 or Core 2, like Core 1B (33–72) [45]. Thus, the oligopeptide repeats are a likely target for recognition by Hsp104 partner chaperones, which facilitates subsequent prion fragmentation. This agrees with earlier observations that the deletion of some of these repeats decreases fragmentation and turns a strong [*PSI*^+^] into a phenotypically weak one, despite preserving strong [*PSI*^+^] structure [54,55].

The finding that some prion structures of Sup35 can be phenotypically silent [45] adds uncertainty to the definition of prion variants. The assay for the discrimination of [*PSI*^+^] variants [41] relies on mutations in Region 1 and thus can only discriminate the variation of Core 1. It is not clear whether presence of Cores 2 to 4, which seem to be nearly phenotypically silent, should be regarded as additional [*PSI*^+^] strain variation. 

### 2.4. On the Equivalence of Sup35 In Vitro Fibrils and In Vivo Prions

Structural studies of the Sup35 amyloid are often performed with Sup35 fibrils that are spontaneously generated in vitro. Fibrils obtained at 4 °C induce mainly strong [*PSI*^+^] variants when introduced to yeast cells and those generated at 25–37 °C induce predominantly weak [*PSI*^+^] variants [56]. Due to this, such fibrils are usually regarded as structurally equivalent to the strong and weak Sup35 prions. Although such equivalence is likely to be correct as a rough approximation, there are some significant concerns as to its complete validity. The first is that some level of structural heterogeneity of the in vitro fibrils and the existence of four different weak [*PSI*^+^] variants potentially correspond to the 25–37 °C fibrils [41]. Secondly, the amyloid fold obtained in vitro can be altered upon propagation in yeast. For example, Ohhashi et al. obtained in vitro Sup35 amyloids with protease-resistant cores at residues 81–148 and 62–144 [43]. When introduced to yeast cells, these amyloids induce strong and weak [*PSI*^+^] variants, VH and VK [41], both of which, as we observed, have an entirely different structure, including the N-terminal protease-resistant Core 1 (2–72), but they lack the original in vitro-generated core [45]. The 4 °C “strong” fibrils show significantly higher fragility than the 25–37 °C “weak” fibrils [56], which agrees with their lower thermal stability [57]. However, the 4 °C fibrils also show an almost five-fold polymerization reduction [56], which, according to the quantitative prion conversion model presented in the same work, poorly agrees with the much lower level of soluble Sup35 in strong [*PSI*^+^] cells.

## 3. Mechanisms for the Fragmentation and Disassembly of Amyloids in Yeast and Animals

### 3.1. Replication of Yeast Prions

**Figure 2:**
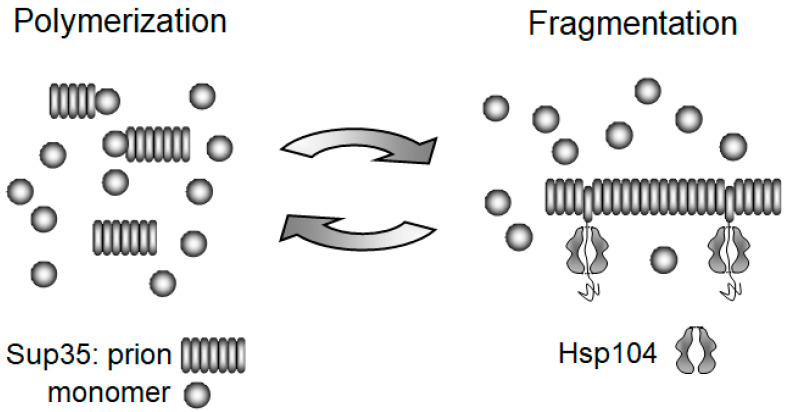
Yeast prion replication cycle [29]. Prion fibrils are multiplied through the action of Hsp104, which randomly extracts protomers from amyloid, thus fragmenting it into smaller pieces. The size of amyloid fragments is restored through growth by accretion of soluble Sup35 molecules. The action of Hsp104 is preceded by, and depends on, binding of the Sis1 and Ssa chaperones [66], which are not shown here.

The study of yeast prions played a crucial role in understanding how prions and amyloids multiply. It was found that the mild overproduction of the Hsp104 chaperone can cure some variants of the [*PSI*^+^] prion. Paradoxically, the lack of Hsp104 has an even stronger effect, eliminating all [*PSI*^+^] variants [58]. Our group offered an explanation for this paradox and the role of Hsp104 [29,59], relying on three earlier observations. Firstly, Hsp104 is able to extract single molecules from the large aggregates of heat-denatured proteins [60]. Secondly, Sup35, in agreement with the amyloid model, forms amyloid fibrils in vitro [61,62]. Thirdly, prion conversion activity correlates with the aggregated, rather than the soluble, form of Sup35 [59,63,64]. We reasoned that if Hsp104 would extract a molecule from the middle of a fibril the same way it extracts them from heat-denatured aggregates, the fibril would split, producing two prion particles from one. This, in fact, completes the prion replication cycle, the other part of which is the growth of Sup35 fibrils by their joining, as well as the conversion of soluble Sup35 molecules (Figure 2). This simple mechanism ensures prion propagation in three ways. Firstly, the inheritance of a prion requires that the prion particles should multiply, not just grow in size, since they are constantly diluted, i.e. reduced in number during cell division. Secondly, the distribution of prion particles to daughter cells is nearly random for smaller particles, while larger ones tend to be retained in the mother cells [65]. Therefore, reliable inheritance requires tens or hundreds of prion particles per cell. Thirdly, since prion conversion occurs at the fibril ends, fragmentation creates new ends and thus accelerates the conversion of soluble proteins to their prion form. We presumed initially that overproduced Hsp104 cures [*PSI*^+^] by dissolving prion fibrils, but, as discussed in Section 4.2, the curing mechanism is likely to be different.

The paradoxical beauty of this mechanism is that none of its parts are likely to have evolved for prion replication. The fibrillar shape is a universal property of amyloids, while the Hsp104 mechanism was most likely developed to cope with amorphous stress-derived aggregates, rather than prions. It is also noteworthy that the replication of yeast prions relies on the Hsp104 action aimed at their destruction.

**Figure 3:**
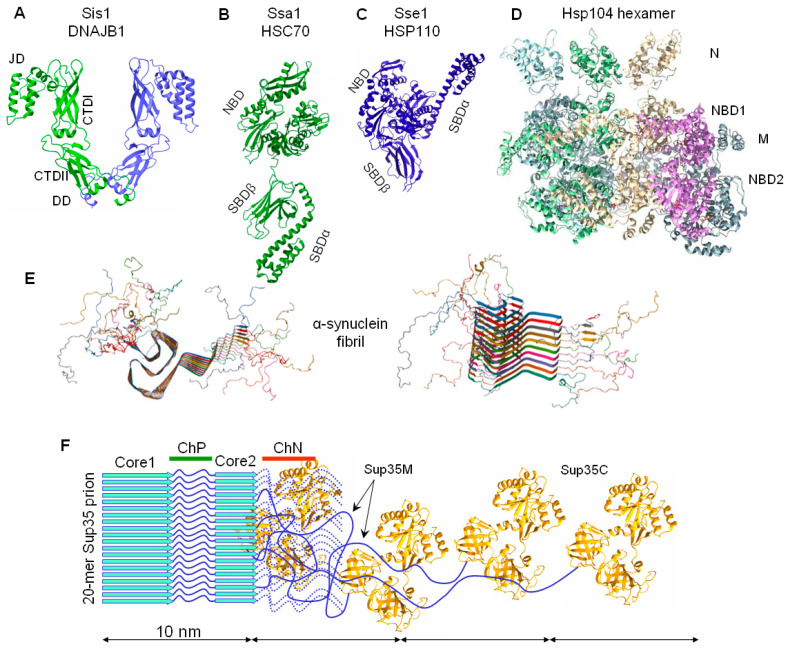
Relative sizes of a α-synuclein fibril, Sup35 prion, and the chaperones involved in their fragmentation. The sizes are drawn to scale. Structures of yeast chaperones are presented, while their mentioned human orthologs are of similar size and structure, such as (**A**) Sis1/DNAJB1 dimer (Sis1: PDB 6D6X+1CG3). JD: J domain; CTDI, CTDII: carboxy-terminal domains I and II; DD: dimerization domain. (**B**,**C**) Ssa1/Hsc70 and Sse1/HSP110 (Ssa1: PDB 2KHO, Sse1: PDB 2QXL). NBD: nucleotide binding domain; SBDα and SBDβ: substrate-binding domain. (**D**) Hsp104 hexamer (PDB 5KNE). N, M: domains N and M; NBD1, NBD2: nucleotide binding domains 1 and 2. (**E**) α-syn fibril (PDB 2N0A). (**F**) Sup35 prion (Sup35C: PDB 1R5B). Sup35 prion structure with two protease-resistant cores is depicted, though in some [*PSI*^+^] variants Core 2 is absent and/or additional structures in the M domain are present. For clarity, only four Sup35 C domains and M domains (blue line) are shown; other copies of the M domain are indicated by dotted lines. ChP—a non-structured protease-sensitive region of the Sup35N domain, presumably used for productive binding of Hsp104 via Sis1 and Ssa chaperones. ChN—the region 129–148 of direct but non-productive Hsp104 binding. The diameter of the amyloid core, presumably corresponding to the Sup35 N domain, is about 8–10 nm [53,68]. The unfolded M domain allows the C domain to be widely offset from the fibril core, thus they form a loose halo with a diameter of 60–65 nm [68]. Notably, the M domain can acquire a folded [53] protease-resistant state, at least in a part of the prion population [45,53].

The proposed mechanism of prion fragmentation by Hsp104 was later confirmed by many studies. In particular, we developed an electrophoretic method to determine the size of amyloid fibrils, and showed that Sup35 prion particles are relatively small and include 10 to 50 Sup35 molecules [44]. Hsp104 is inhibited by a low concentration of guanidinium hydrochloride (GuHCl, 3–5 mM) in the growth medium [67]. Such inhibition increases the size of Sup35 prion particles two-fold per generation, exactly as expected in the case of the complete block of fragmentation [44]. Notably, with such a size, Sup35 fibrils should look quite different from the long fibrils obtained in vitro, since the length of a 20-mer fibril (~10 nm) is about the same as its width (Figure 3). 

It may appear that the prion fragmentation concept is too obvious, but it was not so clear at the time when it was proposed. The discovery of yeast prions was inspired by the human prion concept of Stanley Prusiner [69], but this concept included the heterodimer model for prion replication [70], which considered prions as monomers. In this model, a prion molecule PrP^Sc^ forms a heterodimer with its normal conformer PrP^C^, converts it into PrP^Sc^, and then two PrP^Sc^ molecules dissociate. We concluded that such a process is unlikely, because (1) it cannot occur without an external source of energy and (2) it poorly accommodates the existence of prion strains or multiple alternative conformations of PrP, capable of self-catalytic conversion. In contrast, amyloids are known to form alternative self-catalytic structures (reviewed in [71]). Therefore, we switched to the amyloid model of the prion, which was free from these problems, though initially it did not focus on the multiplication of amyloid particles, which is especially important in the case of prions. We proposed that the multiplication is performed through fragmentation by the Hsp104 chaperone, which simultaneously provides the required source of external energy [29].

### 3.2. The Chaperone-Mediated Fragmentation of Yeast Prions 

While Hsp104 is a key element of the yeast fragmentation mechanism, it does not act alone. For its function, Hsp104 requires the assistance of chaperones belonging to the Hsp40 (called J-proteins, due to their homology to bacterial DnaJ) and Hsp70 (similar to DnaK) families. In particular, Ydj1 (Hsp40) and Ssa-family (Hsp70) proteins are required to solubilize and reactivate denatured luciferase [72]. There are four functionally interchangeable Ssa proteins in yeast, Ssa1 to Ssa4, which we call here, collectively, Ssa. Ssa1 and Ssa2 are constitutively expressed and associate with prions [73], as well as with non-prion Sup35 complexes [74]. Yeast has 13 proteins in the Hsp40 family, which differ in their substrate specificity and their participation in the maintenance of yeast prions. Among them, the key role in prion propagation belongs to Sis1, which is required for the propagation of at least four yeast prions, [*PSI*^+^], [*PIN*^+^], [*URE3*], and [*SWI^+^*] [75,76,77,78,79]. Three other J-proteins are also involved in prion propagation: Ydj1, Apj1, and Swa2 [78,80,81,82]. Current models assume that Sis1 and Ssa proteins act together to recognize prions and present them to Hsp104 for unfolding, which is required for Hsp104 disaggregating activity [66,77].

Hsp104 functions in the form of a ring-like hexamer with a central pore. Hsp104 extracts proteins from amyloid or amorphous aggregates by threading their polypeptide chain through the pore and allowing it to fold properly at the distal end. Hsp104 is a large protein containing two ATPase units, nucleotide binding domains (NBD) 1 and 2. The movement of the polypeptide chain through the Hsp104 pore is achieved by the sequential contraction of Hsp104 protomers at the expense of ATP hydrolysis by NBD1, while NBD2 appears to be required for the assembly of the hexamer [83]. For each ATP molecule spent by NBD1, Hsp104 advances along the peptide chain by two residues [84,85,86]. A frequent scenario is that Hsp104 starts unfolding from a loop in the middle of a protein. In this case, Hsp104 threads two polypeptide strands simultaneously [86], thus doubling its energy efficiency.

The mechanism of Hsp104 inhibition by GuHCl has also been elucidated. It was shown that in the bacterial Hsp104 homolog ClpB, GuHCl binds specifically to the active site of NBD1 [87]. GuHCl strengthens the Hsp104 M domain/NBD1 interaction, stabilizing Hsp104 in a repressed conformation, abrogating Hsp70 cooperation and inhibiting continuous ATP turnover by NBD1 [88].

Hsp104 is a very powerful, but also costly, molecular machine: under non-stressing conditions, cells lacking Hsp104 grow noticeably faster. When wild-type and *hsp104Δ* cells were grown together in a competition assay, the proportion of wild-type cells decreased from 50% to 20% during two days of growth. However, this proportion remained constant for the next two days [89], which suggests that the cells remaining after two days were not inhibited in growth. It is possible that the starting wild-type cells were heterogeneous for the Hsp104 level and there was a fraction of cells with low Hsp104 not inhibited in growth.

Different Hsp104 substrates may vary in their mechanical strength and, therefore, may require different mechanical forces to unfold them. By mixing functional and nonfunctional Hsp104 subunits, DeSantis et al. showed that “easy” substrates like disordered heat-denatured aggregates of luciferase do not require the cooperation of Hsp104 subunits and can tolerate a high proportion of mutant Hsp104. A stronger substrate, like Sup35’s “strong” in vitro formed fibrils, requires the cooperation of three sequential subunits. The disassembly of the most mechanically stable “weak” Sup35 fibrils requires the cooperation of all six Hsp104 subunits and is the least tolerant to the presence of nonfunctional Hsp104 [90].

### 3.3. Amyloid Fragmentation in Animals

Hsp104 orthologs are present in all kingdoms of life, in prokaryotes (ClpB) and eukaryotes, except for animals (metazoa). Hsp104 was present in the last common ancestor of animals and choanoflagellates, but was lost in the last common ancestor of animals. The mitochondrial Hsp104 analog, Hsp78, was lost simultaneously [91], and so the loss does not appear accidental. The authors relate the loss to the simultaneous disappearance of some Hsp104 clients among metabolic enzymes [91], but one can also suggest that mobility gives animals more opportunities to avoid stressful conditions requiring Hsp104, the costly machine with 12 ATPase units that may be disadvantageous in the absence of stress.

Searches for disaggregase activity in animal cells revealed that even without Hsp104, the Hsp40-Hsp70-mediated mechanism can result in moderately efficient disaggregase activity. It shows a strong dependence on another participant of this mechanism, the Hsp110 (APG-2) chaperone, which is a nucleotide exchange factor for Hsp70 [92,93,94]. In yeast, the role of analogous factors like Sse1/2 is also important, but it becomes more evident in animals, which lack Hsp104 [94].

This mechanism was studied in fine detail by Wentink et al. using α-syn amyloids as a target [95]. In humans, this mechanism includes the J protein DNAJB1, an ortholog of yeast Sis1, the constitutive human HSP70 (HSC70, also known as HSPA8) and HSP110 (APG-2, also known as HSPH2 and HSPA4). First, these authors showed that the DNAJB1 binding affinity to α-syn fibrils is much higher than to the monomer α-syn and that binding is strongly required for DNAJB1 dimerization. The maximal binding density proved to be about one DNAJB1 dimer to ten α-syn protomers. To envision the interaction of chaperones with amyloids, it is helpful to keep in mind a characteristic property of amyloids: the very close spacing of protomers, equal to 0.48 nm. The low proportion of DNAJB1 is then not surprising, since its dimer size is about 10nm, which corresponds to the length of a 20-protomer fibril (Figure 3). DNAJB1 stimulates HSP70 binding to fibrils up to ten-fold, at a proportion of up to five HSP70 molecules per one DNAJB1. Thus, the maximal binding proportion of HSP70-to-α-syn is one for every two protomers. This appears to be a very high density of HSP70, considering the size of HSP70 molecule (Figure 3). Such dense packing of HSP70 molecules should cause steric pushing and create forces that break the amyloid fibrils. This mechanism of amyloid disassembly is termed entropic pulling. 

The way HSP110 helps this mechanism proved to be very unexpected. Due to its large size of 94 kDa, HSP110 can reach and release only those HSP70 molecules, which are not clustered. In this way, HSP110 locally concentrates HSP70, thus increasing the efficiency of entropic pulling. BAG1 is another nucleotide exchange factor for HSP70, but it is a small molecule with a size of 31 kDa. BAG1 does not stimulate amyloid disassembly, but, remarkably, it starts doing so when its size is artificially increased [95].

### 3.4. Yeast Prions Based on Putative “Soft” Amyloids or Non-Amyloid Structures

Two basic properties of amyloids seem to be essential for them to exhibit prion properties: the ability for the indefinite autocatalytic propagation of altered protein folds, and the filamentous shape that is important for prion replication by fragmentation. Accordingly, the existence of a prion related to a heritable structural change in a protein, which is not of an amyloid type, seemed unlikely. Nevertheless, such prions were discovered in yeast. It is worth noting that one prion formally matching such a definition is known: it is an artificial prion [β], which is related to the proteolytic self-activation of protease Prb1 [96]. However, in this review, we consider only prions that are based on the alterations of spatial structure that do not modify the primary structure of the protein.

Study of the [*GAR*^+^] prion revealed that it is related to some non-amyloid structural alterations of the Pma1 and/or Std1 proteins. [*GAR*^+^] shows some properties typical of amyloid prions: it appears with a greatly increased frequency upon the overproduction of Pma1 or Std1, it is transmissible by cytoduction (the transfer of cytoplasmic genetic elements between yeast cells through an abortive mating procedure, in which nuclear fusion is prevented by a mutation blocking karyogamy, usually *kar1* [97]), and it shows a barrier in prion transmission between closely related species. However, no change in the aggregation state of either Pma1 or Std1 was detected [22].

In 2016, a groundbreaking study revealed a large swath of 46 yeast prions, most of which are not based on SDS-resistant aggregates [23] (one of the main hallmarks of amyloids [17,44]). These prions were discovered by monitoring heritable alteration of yeast phenotypes (sensitivity to various salts and antibiotics) after transient overproduction of the respective proteins. Their prion state is readily transmitted through spheroplast “protein” transformation with [*PRION*^+^] lysates treated with DNase and RNase. In some, but not all, cases, a change of the aggregation state is evident by the microscopic observation of fluorescently labeled prion molecules. Most of the new prion proteins have large intrinsically disordered regions, but these are not rich in glutamine and asparagine as is typical of amyloid-based yeast prions. The new prions show a different dependence on chaperones.

Eleven of these 46 prions are curable by GuHCl and therefore require Hsp104 activity like the amyloid-based yeast prions. However, these 11 prions did not form detergent-resistant polymers, which allowed the authors to conclude that they do not form amyloids. However, such a conclusion could be incorrect. While all known yeast prion amyloids are insoluble in SDS at room temperature [17,44], this does not necessitate that all amyloids must show such a property. For instance, yeast Pub1 forms both SDS-insoluble and soluble amyloids in vivo, though the latter are insoluble in a milder ionic detergent, sarcosyl [98]. SDS-soluble amyloids have also been observed for several proteins related to human amyloidosis, such as FUS and hnRNPA2 [99,100], as well as for yeast Pbp1, which is an ortholog of human ataxin-2 and a binding partner of Pbp2, one of the newly identified “non-amyloid” prions [101].

The remaining 35 Hsp104-independent prions were tested for their requirements in other chaperones. Nineteen prions proved to be curable by the transient expression of the Ssa1-K69M mutant protein, which dominantly inhibits Ssa function [102]. This subset includes the three best-studied members of the non-amyloid prion cohort: [*GAR*^+^], [*BIG*^+^], and [*SMAUG*^+^]. [*BIG*^+^], the prion form of pseudouridine synthase (Pus4), accelerates cell growth and reduces the replicative lifespan. [*SMAUG*^+^], the prion form of the Vts1 protein, orchestrates gene expression by regulating mRNA decay. [*SMAUG*^+^] was proposed to have a transmissible agent in the form of a hydrogel that can seed the conversion of monomeric Vts1 molecules into a gel-like state in vitro. This hydrogel is soluble in ionic detergents even at their low concentrations and shows no signs of filamentous structures [103]. 

**Figure 4:**
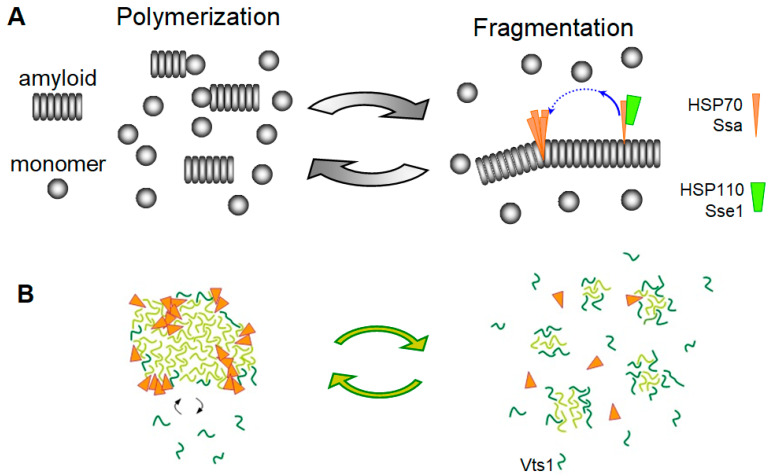
Amyloid and prion fragmentation in the absence of Hsp104. (**A**) “Entropic pulling” amyloid fragmentation by HSP70 and HSP110. Hsp110 preferentially removes non-clustered HSP70 molecules from the amyloid, thus promoting formation of HSP70 clusters, where entropic pulling can create sufficient force to fragment amyloid. Hsp40 that initiates Hsp70 binding is not shown. Originally described for α-syn [95], this model could also work for yeast prions based on soft amyloid structure and independent of Hsp104, but dependent on Ssa. (**B**) Proposed fragmentation by Ssa of yeast prions based on hydrogels like [*SMAUG*^+^]. Vts1 monomers joining hydrogel are shown in dark green.

Thus, it may be proposed that the yeast Hsp70 Ssa proteins can fragment and can thus replicate prions made of either amyloids with a soft SDS-soluble structure like Pbp1, or hydrogels like Vts1, using the same “entropic pulling” mechanism described above for the animal Hsp70 and its co-chaperones [95]. Amyloids are one-dimensional crystals (i.e., their basic unit is repeated in just one dimension), and such topology was important for formulating the fragmentation model [29]. Though hydrogels are three-dimensional, this model could be extended to 3-D structures (Figure 4).

Importantly, the intrinsically disordered regions present in the novel yeast prions are also found in homologous human proteins. Some of these human proteins were expressed in yeast as GFP fusions and exhibited a heritable aggregation induced by the overproduction of these proteins. These included Ipo11 (a homolog of yeast Kap120), Pold3 (a homolog of Pol32), [23] and hSMAUG (a homolog of Vts1) [103]. This suggests that the corresponding epigenetic elements may also act in humans and animals.

## 4. Elimination of Prions and Amyloids by Hsp104 and Related Chaperones

### 4.1. Overproduced Hsp104 Acts Differently at the Two Levels of Yeast Prion Structure

Regrettably, many studies of yeast prions do not differentiate between prion fibrils, prion aggregates, and propagons. We observed that the Sup35 prion particles represent higher order aggregates composed of a number of relatively short fibrils made up of 10–50 Sup35 protomers, as well as some additional proteins [44]. A propagon is a genetic entity defined by the assay developed by Cox et al. [104] where the multiplication of prion particles is supposed to be blocked by GuHCl and these particles are allowed to segregate to individual cells, while an original cell divides for several generations and forms a small colony. This colony is then spread to single cells on a plate that lacks GuHCl and the propagons are then counted as the number of colonies retaining prions. Thus, propagons should be equivalent to prion higher order aggregates, provided that these aggregates neither merge nor split during their growth in the presence of GuHCl. GuHCl fully blocks the fragmentation of prion fibrils [44], but the same was not strictly shown for aggregates. Propagons are clearly different from prion fibrils. Strong [*PSI*^+^] fibrils include, on average, about 20 protomers [44]. A yeast cell with strong [*PSI*^+^] variants contain about 80,000 Sup35 molecules [105], 4000 fibrils, and 200–1000 propagons [65,106]. Thus, each propagon from a strong [*PSI*^+^] contains, on average, 4 to 20 Sup35 fibrils. While fibrils have a strong amyloid structure that is insoluble in SDS, higher order aggregation is based on some weaker interactions that are sensitive to SDS [44].

**Figure 5:**
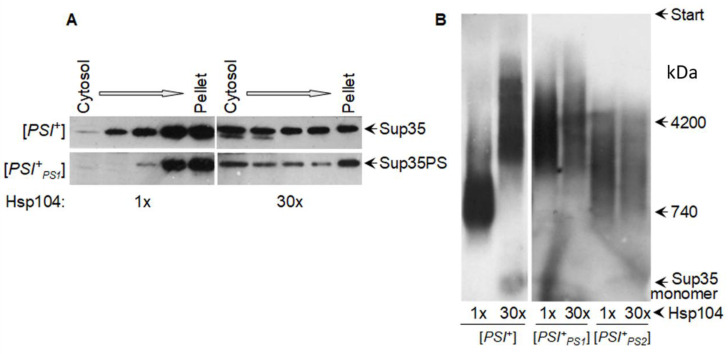
Overproduced Hsp104 disassembles Sup35 prion aggregates, but not fibrils. Hsp104 was either endogenous, or produced from a multicopy plasmid under control of the native promoter (~30-fold excess). (**A**) Sup35 aggregates of yeast lysates fractionated by centrifugation. Sup35PS, [*PSI*^+^_PS1_], and [*PSI*^+^_PS2_] relate to Sup35 with prion domain from yeast *P. methanolica* and M and C domains from *S. cerevisiae* and its two prion variants. (**B**) SDD-AGE of Sup35 prion fibrils. (**A**,**B**) Western blot staining for Sup35NM. Data reproduced from previous research [107] (**A**), [44] (**B**).

Our old experiments revealed paradoxical details regarding the effect of overproduced Hsp104 (a multicopy of *HSP104* on a glucose medium) on Sup35 prion aggregates. The size of prion aggregates (as revealed by centrifugation) substantially decreased, while the size of prion fibrils (as assayed by agarose gel electrophoresis) increased or remained constant (Figure 5). These effects were observed for strong [*PSI*^+^] variants and artificial prions with the Sup35 N domain from the yeast *Pichia methanolica* [44,107], while weak [*PSI*^+^] variants could not propagate in such conditions [45]. The differential fate of aggregates and fibrils was not understood previously, but it can be explained now with the aid of later observations. We observed that the Sup35 N and M domains in their prion state contained both amyloid structured and unstructured regions [45]. The latter are likely to mediate the aggregation of prion fibers either directly or through chaperones, since the aggregation is sensitive to Hsp104. The fibril-fragmenting activity of Hsp104 is likely to be restricted not by Hsp104 itself, but by the limited amounts of Sis1 and Ssa that are able to bind to a fibril due to the very close spacing of protomers in amyloids (Figure 3). The proportion of Ssa1 and Ssa2 proteins to Sup35 observed in ex vivo [*PSI*^+^] prions is about 1:2 [73], which is similar to the maximal value observed for α-syn amyloids [95]. Thus, it appears that Sup35 prion fibrils are already saturated with Ssa, since they have the same 0.48 nm protomer spacing as α-syn amyloids. The Hsp104 fragmenting activity cannot be increased since it depends on the amount of prion-bound Ssa, which also cannot be increased. This could explain why the size of the Sup35 fibrils, in many cases, does not change in response to the strong overproduction of Hsp104. The interactions observed between fibrils in the higher-order aggregates do not have such restrictive architecture and, thus, can be responsive to an increase in Hsp104.

### 4.2. Elimination of Yeast Prions by Hsp104

Regarding the attempts to disassemble amyloids in animal models with the help of Hsp104 that was described in the last chapter, it is of interest to consider the Hsp104-mediated curing of prions in yeast. The curing data are paradoxical. Endogenous Hsp104 can cure a high proportion of [*PSI*^+^] variants that emerge in the presence of the weakened Hsp104 mutant, T160M [42,108], and mildly overproduced Hsp104 can cure some [*PSI*^+^] variants [58], but some other variants are not cured even at high Hsp104 overproduction [45].

The data on prion curing by strongly overproduced Hsp104 are contradictory. Though initially it appeared that Hsp104 overproduction should proportionally increase its prion fragmenting and disaggregating activities [29], this is apparently not so. It is often stated that [*PSI*^+^] may be the only yeast prion that can be efficiently cured by excess Hsp104, since [*URE3*] and [*PIN*^+^] are resistant to such impacts [109,110]. However, some level of [*URE3*] curing has been shown in later work [111]. Among [*PSI*^+^] variants, weak ones are readily cured by excess Hsp104, while strong [*PSI*^+^] variants are more resistant. This difference depends somewhat on the specifics of Hsp104 overproduction. When the *HSP104* gene was expressed from a multicopy 2-micron plasmid (this results in about 30-fold Hsp104 overproduction [112]), in the testing of eight weak and seven strong [*PSI*^+^] isolates, all the weak ones were fully cured, while all strong ones were fully resistant [45]. Production under a low copy inducible *GAL1* promoter results in a similar 20–40 fold Hsp104 excess, but it cures a strong [*PSI*^+^] variant almost completely in ten generations of growth [106]. The difference in the curing of strong [*PSI*^+^] variants appears to depend on the carbon source (galactose versus glucose) [45]. We also observed (unpublished data) that the absence or the malfunction of mitochondria allows the complete curing of strong [*PSI*^+^] variants by the multicopy of *HSP104* on glucose. 

The easier curing of weak Sup35 prions appears paradoxical, since they should be less efficiently recognized and disassembled by chaperones compared to the strong ones, as suggested by their larger fibrils [44] and higher mechanical strength [56,90]. Greene et al. proposed that the curing of Sup35 prions mainly occurs through the Hsp104-mediated trimming of prion fibers from the ends. The extraction of Sup35 protomers from the prion ends does not generate new prion particles and it is likely to proceed relatively fast, since the ends of a fiber could be more accessible and the terminal protomers are easier to unfold as they are joined to just one other protomer, rather than two. These authors tried to confirm this idea by microscopic observation of the dissolution of the aggregates of GFP-labeled prion particles, after the start of Hsp104 overproduction, in several reviewed works [113]. However, this approach does not appear adequate for the question. The visible prion–GFP foci represent large aggregates, including many prion fibrils [44]. As we already noted, overproduced Hsp104 can disassemble these aggregates into smaller particles, down to single fibrils [107] that are undistinguishable from monomers by light microscopy, without decreasing the size of these fibrils [44]). The best way to observe trimming would be to monitor the size of prion fibrils by agarose electrophoresis, called SDD-AGE [44], but this was not done. 

Serio et al. proposed that Sup35 prion fibrils smaller than a certain threshold size may be unstable and would be dissolved, and that such a threshold is higher for weak prions. Hsp104 action not just at, but also near, prion fiber ends would act as a trimming process, generating no new prion particles; the higher the minimal size of weak Sup35 prions could explain their easier curing by Hsp104. However, while a mathematical model of this effect was provided, the experimental evidence in support of this idea [114] does not appear sufficient. It is also unclear why weak Sup35 prions, which are mechanically sturdier than strong prions [56] and less well-recognized by chaperones, require more protomers to stabilize their minimal particles. 

Another more convincing group of data, although still partly contradictory, relates the [*PSI*^+^] curing by overproduced Hsp104 to prion malpartition, rather than dissolution. The initial observation was that Hsp104 can bind directly to the Sup35 M domain region 129–148, which is important for curing, since Sup35 prions lacking this region are not cured by Hsp104 overproduction [33].This Hsp104 binding is independent of Hsp40 and Hsp70 and, in relation to this, nonproductive [66]. In line with this, in vivo Hsp104 shows two types of binding to Sup35 prions: one is labile, Hsp70-dependent, and exhibits a free exchange of Hsp104 with the pool of monomers; the other is a stable binding to the Sup35 M region that shows little exchange [115]. The latter could be termed a direct and non-productive binding. Such a binding should sterically interfere or compete with the binding of different chaperones belonging to the productive fragmentation pathway, due to the relatively small space where such interactions occur (Figure 3), and this could thus reduce prion fragmentation. In agreement with this, overproduced Hsp104 can substantially increase the size of strong Sup35 prion fibrils (Figure 5B) [44]. In contrast, Ness et al observed that Hsp104 overproduction under a low copy *GAL1* promoter did not impair fragmentation and did not alter Sup35 oligomer size. In their setup, the curing of strong [*PSI*^+^] variants, which occurred at a rate of about 10% per generation, was shown to be due to prion retention in a proportion of mother cells. These authors proposed that Hsp104 mediated prion binding to some subcellular structures, thus causing their malpartition [106]. Regrettably, such a study was not made for weak [*PSI*^+^] variants where curing would be more pronounced.

[*PSI*^+^] variants can also be cured with moderate efficiency by short-term heat shock. Such a shock increases the proportion of Hsp104 to Ssa proteins, and the prion curing also occurs through the asymmetric segregation of the prion [116,117]. 

Notably, the weak [*PSI*^+^] variant shows a larger proportion of stable Hsp104 binding to the Sup35 M domain [115], which can explain the easier curing of weak [*PSI*^+^] variants. In turn, the more efficient direct binding to weak Sup35 prions could be explained by our recent data. The Hsp104 target region of Sup35 is 129-148, which coincides well with amyloid Core 3 (124–153) that forms in the majority of strong [*PSI*^+^] variants but is rarely found in weak [*PSI*^+^] variants [45]. It is reasonable to assume that Hsp104 can bind the target region in its unfolded state, but not while in an amyloid fold, so non-productive Hsp104 binding is less likely in strong [*PSI*^+^] variants.

On the other hand, the productive binding of Hsp104 through Sis1 and Ssa should be more efficient in strong [*PSI*^+^] variants. These chaperones bind to the unfolded regions of the Sup35 N domain, rich in tyrosine, which stimulates fragmentation best [57,118]. Such regions are smaller in weak Sup35 prions due to the presence of Core 2 (~90–123) amyloid structure that is rare, or less pronounced, in strong [*PSI*^+^] variants [45]. Thus, the reduced productive and increased non-productive Hsp104 binding could define the easier curing of weak [*PSI*^+^] variants by overproduced Hsp104.

Thus, despite some experimental discrepancies, the only confirmed mechanism of [*PSI*^+^] curing by overproduced Hsp104 is the inefficient partitioning of propagons in cell divisions, due to either reduced prion fragmentation, or Hsp104-mediated prion anchoring. Prion curing through its dissolution currently lacks sufficient evidence. Curing by normal Hsp104 levels of the prions generated in the presence of mutant Hsp104 is of great interest in this respect, but its mechanism has not been studied in detail.

### 4.3. The Therapeutic Potential of Protein Disaggregases

While the human Hsc70 system can efficiently disaggregate toxic oligomers and short amyloid fibrils, its activity against large, less toxic amyloid aggregates is severely impaired [119]. Yeast Hsp104 is a much more powerful disaggregase than the Hsp70 (Ssa)-Hsp40 (Sis1) system alone [94], but animals have no Hsp104 homolog [91]. This raised hope that Hsp104 can disaggregate pathological amyloids if reintroduced to animal cells [120,121]. Luckily, Hsp104 can efficiently collaborate with the animal disaggregation machinery and strongly improves the reactivation of heat-denatured luciferase [94,122]. In vitro, Hsp104 can dissolve fibrils associated with human diseases: amyloid β, α-syn, prion protein, tau, amylin, and polyglutamine [90,122,123]. In animal disease models, Hsp104 reduces polyglutamine toxicity in *Caenorhabditis elegans*, fly, and rodent models [124,125,126,127]. In a rat model of Parkinson’s disease, Hsp104 reduced the formation of phosphorylated α-syn inclusions and prevented nigrostriatal dopaminergic neurodegeneration [122].

However, the activity of yeast Hsp104 against pathological aggregates can be insufficient even at high levels of Hsp104 [90]. This prompted attempts to enhance Hsp104 disaggregation activities, and this was, surprisingly, achieved through minor changes and even single missense mutations [128]. Potentiated Hsp104 variants have been developed and are capable of suppressing toxicity associated with α-syn, TDP-43, and FUS in yeast [129,130,131,132,133]. Many such potentiating mutations were found in the regulatory coiled-coil middle (M) domain of Hsp104 (residues 411–538), which mediates interactions of Hsp104 with Hsp70. Hsp104 potentiation often correlates with the destabilization of the M domain. However, some of these mutations show off-target toxicity. To overcome this problem, scanning mutagenesis of the M domain was performed [134], as well as mutagenesis of Hsp104 NBD1 and NBD2 [129,133], which allowed the isolation of non-toxic potentiated Hsp104 mutants. The screening of a cross-kingdom collection of Hsp104 homologs in yeast proteotoxicity models revealed that prokaryotic ClpG reduces TDP-43, FUS, and α-syn toxicity, whereas prokaryotic ClpB is ineffective. The latter is not surprising, since Reidy et al. showed that bacterial ClpB did not properly interact with yeast chaperones and required its bacterial partner chaperones to function [135]. Distinct eukaryotic Hsp104 homologs were uncovered that selectively antagonized α-syn condensation and toxicity in yeast and dopaminergic neurodegeneration in *C. elegans*. Surprisingly, this therapeutic variation does not manifest as enhanced disaggregase activity, but rather as an increased passive inhibition of the aggregation of specific substrates [136]. An Hsp104 variant that is efficient against TDP-43, α-syn, and polyglutamine, that lacked toxicity, was obtained from the thermophilic fungus *Calcarisporiella thermophila* [137]. 

While animals lack the mitochondrial Hsp104 homolog, Hsp78, disaggregation in mitochondria can be performed by Skd3, another chaperone of the AAA+ family. Skd3 shows a homology with bacterial ClpB, but has only one NBD. Mutations in Skd3 that reduce its disaggregating activities are associated with 3-methylglutaconic aciduria, a severe mitochondrial disorder. Thus, Skd3 is a potent mitochondrial protein disaggregase which can be used for treating mitochondrial protein aggregation [138].

AAA+ proteins that do not belong to the Hsp104 family can also be used to counteract toxic protein misfolding in animals. One such protein is archaeal PAN, an unfoldase homologous to the eukaryotic proteosomal 19S particle. PAN associates with the 20S catalytic particle and unfolds substrates before their degradation [139]. A PAN variant was recently constructed with a C-terminal FLAG epitope tag (PANet), which impedes PAN interactions with the 20S proteasome, but does not affect unfolding. The expression of PANet in rod photoreceptors in a mouse model of retinopathy mitigates photoreceptor degeneration caused by protein misfolding without causing significant side effects [140]. Thus, protein disaggregases of the AAA+ family have significant therapeutic potential. 

It is important to keep in mind that the disaggregation of amyloids can have both positive and negative effects. Negative consequences might occur when large amyloids are broken into smaller pieces, but the latter are not efficiently destroyed. This resembles the situation with yeast prions, which are propagated by Hsp104. In animals, amyloids of smaller size are (1) often more toxic and (2) have a much higher potential for a prion-like spread between cells and tissues. Such problems were highlighted recently by Tittelmeier et al. who observed that reducing disaggregation in *C. elegans* by knocking down Hsp110 caused a beneficial decrease of the amounts of toxic α-syn species and a reduction in the intercellular propagation of α-syn aggregates. A similar treatment decreased the amount of polyQ aggregates and their toxic effects in another *C. elegans* model [141]. This suggests that the optimal disaggregation activity would be one that destroys small aggregates, but cannot cope with large ones. Humans have three NEFs for Hsp70, which stimulate the entropic pulling effect to a different extent [95]. Possibly, optimal activity might be achieved by adjusting levels of these NEFs. 

The described data raises certain hopes and reveals some fundamental problems. The most optimistic aim would be the complete dissolution of amyloids, but this has never been shown, even in yeast. Still, some hope comes from observations in yeast where prions that appear in the presence of weakened Hsp104 are less resistant to Hsp104, while human amyloids that appear in the absence of Hsp104 yeast prions can be cured through retention in the mother cell, but such a scenario is not valuable for multicellular organisms, where most cells do not actively divide. However, they possess multiple mechanisms for the intercellular movement of amyloids (reviewed in previous research [142]) and these are likely to be affected by factors, which influence prion retention in dividing yeast cells.

A central technical problem would be to deliver Hsp104 to every cell or extracellular location containing an amyloid, which does not seem currently feasible. Finally, a tool able to disassemble all amyloids could also disassemble functional amyloids, and, in particular, amyloids that are involved in long-term memory. 

Nevertheless, disaggregases of the Hsp104 type can alleviate the symptoms of amyloidoses in some animal models. Although there might not be a single ideal disaggregase, different agents tailored for each type of amyloidosis might be possible. In any case, the most promising strategy would be to adjust disaggregases so that they disassembled more toxic, but less resistant, small amyloids, while leaving less toxic and more resistant larger aggregates intact.

## Data Availability

Not applicable.
